# Supplementary material for: Broadly neutralizing antibody induction by non-stabilized SARS-CoV-2 Spike mRNA vaccination in nonhuman primates
Source: bioRxiv. 2023 Dec 19:2023.12.18.572191. Preprint. [Version 1] doi: 10.1101/2023.12.18.572191 (PMC10769253; doi:10.1101/2023.12.18.572191)
Supplement: Supplement 1 [file media-1.pdf]

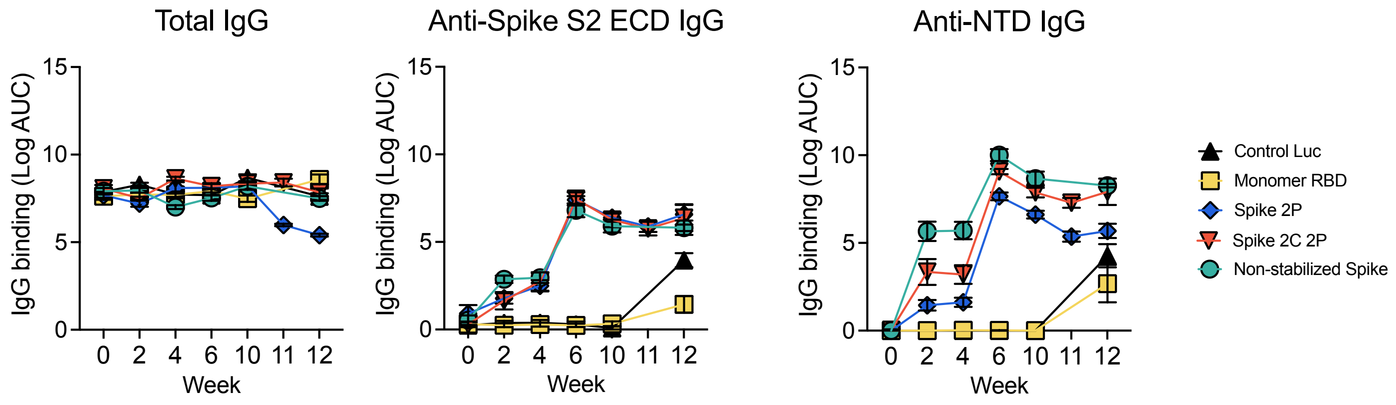

Figure S1. Vaccine-induced SARS-CoV-2 specific IgG binding titers. Total serum IgG binding magnitude to Spike 2P (S-2P), anti-Spike S2 ECD (ectodomain) and anti-NTD IgG were tested by ELISA and shown as log area under the curve (logAUC). Symbols indicate the group mean value ± SEM of three replicates.

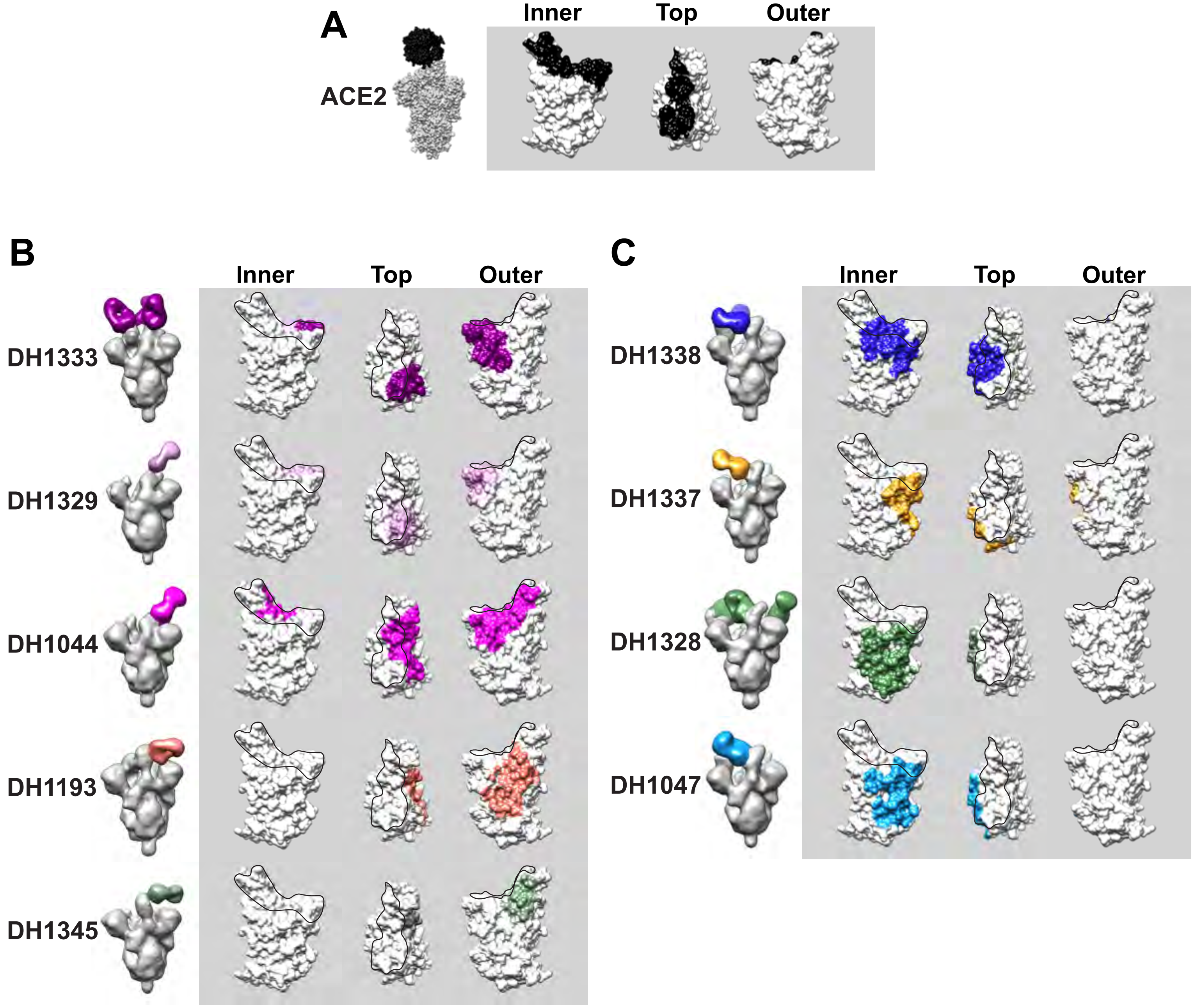

Figure S2. Comparison of ACE2, macaque nAb, and human nAb binding footprints on SARS-CoV-2 RBD. Antibody binding footprint was determined with 3D reconstruction of NSEM images of ACE2 or antibody bound to SARS-CoV-2 Spike. Spike is shown in gray with antibody Fab shown in different colors. The binding footprint on RBD for ACE2 or the specified antibody is shown to the right of the NSEM 3D reconstruction. (A) Structure of ACE2 (black) bound to SARS-CoV-2 Spike (gray). The ACE2 binding footprint is highlighted black on the RBD monomer to the right of the structure. (B,C) Binding footprint comparison for (B) outer and (C) inner domain antibodies. Human nAbs DH1044, DH1193, and DH1047 are shown for comparison to macaque antibodies (DH1333, DH1329, DH1345, DH1338, DH1337, and DH1328).

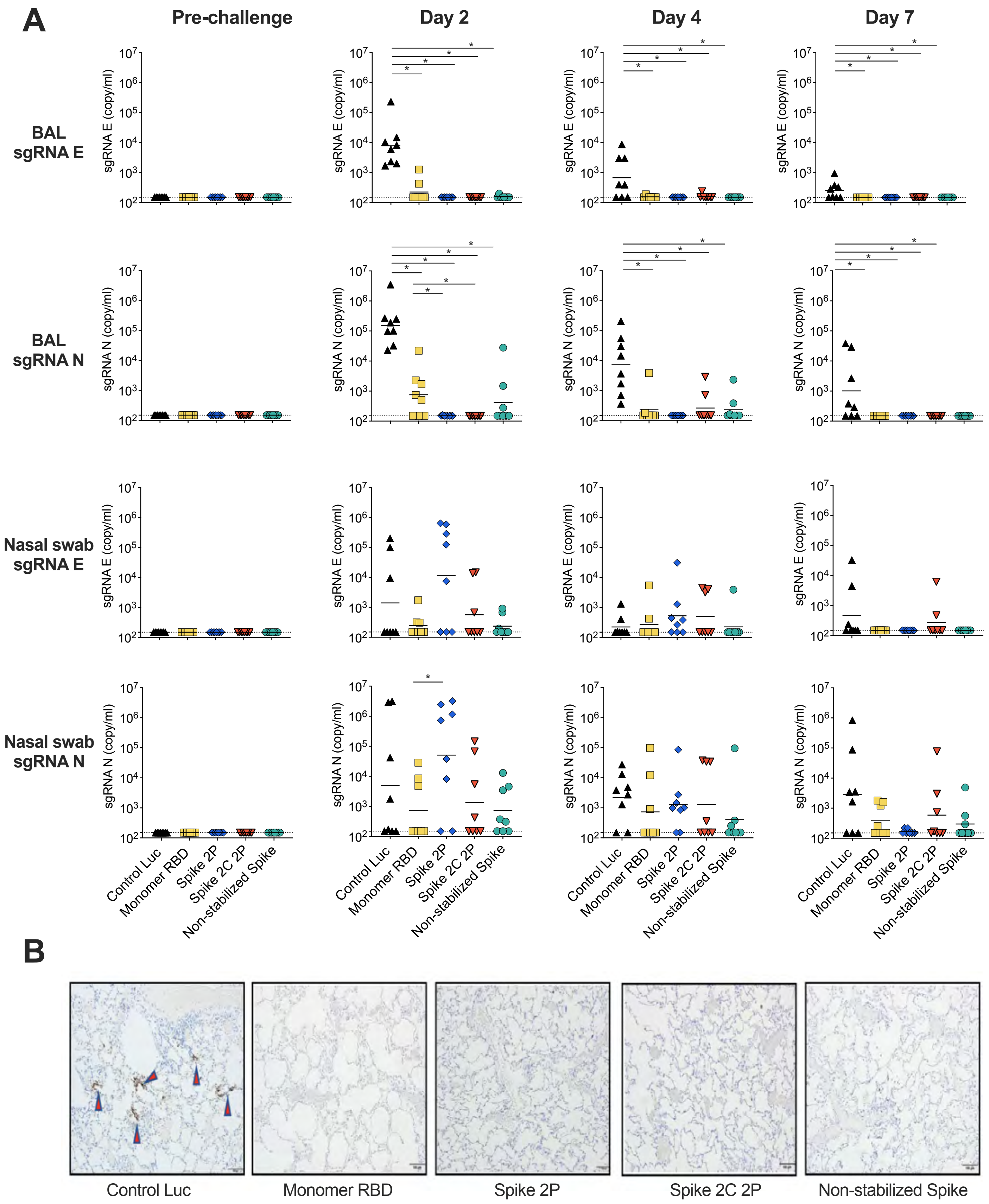

A
Pre-challenge
Day 2
Day 4
Day 7
BAL sgRNA E
BAL sgRNA N
Nasal swab sgRNA E
Nasal swab sgRNA N
sgRNA E (copy/ml)
sgRNA N (copy/ml)
Control Luc
Monomer RBD
Spike 2P
Spike 2C 2P
Non-stabilized Spike
B
Control Luc
Monomer RBD
Spike 2P
Spike 2C 2P
Non-stabilized Spike

Figure S3. mRNA-LNP vaccination prevents virus replication in the lower respiratory tract after intranasal and intratracheal SARS-CoV-2 challenge in macaques. (A) SARS-CoV-2 envelope gene (E gene) sgRNA and nucleocapsid gene (N gene) sgRNA in bronchoalveolar lavage (BAL) and nasal swab samples were quantitated pre-challenge, and on Day 2, 4, and 7. (B) A representative image of nucleocapsid antigen staining from each group of mRNA-LNP vaccinated macaques is shown. All images are shown at 10x magnification. Scale bars, 100 μm.

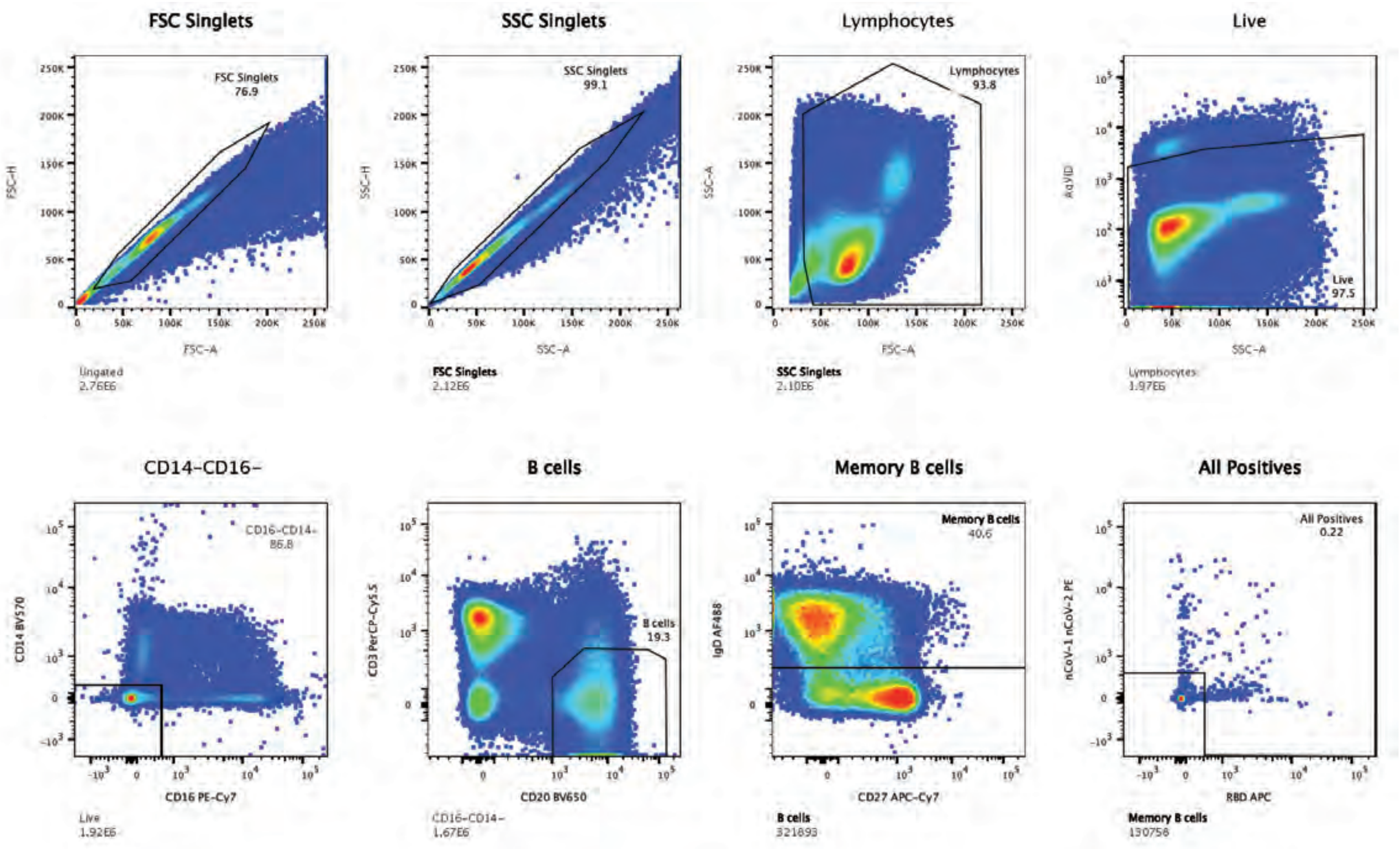

Figure S4. Flow cytometric gating strategies for antigen-specific B cell isolation. The gating strategy to identify SARS-CoV-2 full length spike and RBD-specific memory B cells.

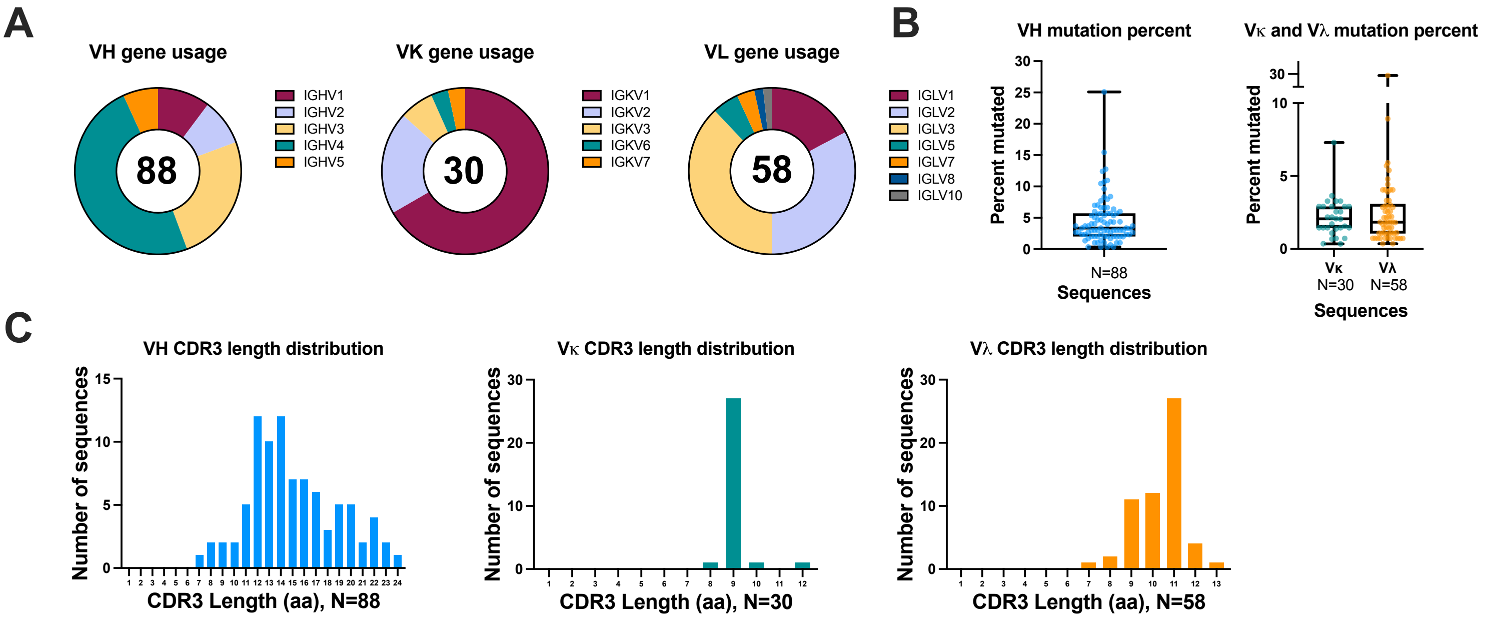

D

Antibody ID	VH gene	VH CDR3 length	VH mutation %	VL gene	VL CDR3 length	VL mutation %
DH1323	IGHV1-c*01	16	3.020	IGKV1-b*06	9	1.460
DH1334	IGHV3-x*04	17	12.752	IGLV3-j*01	10	0.738
DH1340	IGHV4-j*02	16	2.685	IGLV2-j*15	7	0.738
DH1341	IGHV4-m*02	13	6.579	IGLV1-i*02	11	2.166
DH1330	IGHV2-a*02	16	1.329	IGKV1-g*04	9	0.730
DH1324	IGHV4-j*02	17	2.349	IGKV1-r*01	12	1.460
DH1338	IGHV4-j*02	21	1.678	IGKV3-b*19	9	3.650
DH1335	IGHV4-b*01	15	4.362	IGLV3-j*05	11	1.107
DH1333	IGHV3-m*05	15	2.961	IGLV7-c*01	10	0.714
DH1325	IGHV4-n*01	14	2.658	IGLV2-j*15	11	3.321
DH1329	IGHV1-c*01	16	4.027	IGKV3-d*05	9	1.460
DH1328	IGHV4-n*03	23	1.993	IGKV1-g*04	9	0.365
DH1345	IGHV5-a*04	12	2.349	IGLV1-a*03	11	1.805
DH1342	IGHV4-n*03	14	6.645	IGLV3-e*01	11	1.107
DH1336	IGHV4-e*01	14	7.718	IGKV2-g*01	9	2.076
DH1326	IGHV2-b*04	22	1.993	IGLV2-j*16	12	4.059
DH1337	IGHV4-e*01	20	5.705	IGLV1-i*02	12	1.083
DH1339	IGHV4-j*02	14	3.356	IGLV2-d*07	10	8.929
DH1331	IGHV3-j*02	14	2.961	IGLV2-j*15	11	2.214
DH1343	IGHV5-a*03	16	1.007	IGLV1-a*01	11	1.444
DH1332	IGHV3-j*02	13	0.987	IGLV3-j*05	11	3.321
DH1327	IGHV3-k*04	10	2.303	IGKV3-b*19	8	2.190
DH1344	IGHV5-a*04	11	2.013	IGLV1-a*01	12	1.083

Figure S5. Monoclonal antibody isolation from wildtype SARS-CoV-2 spike mRNA-LNP vaccinated rhesus macaques. (A) Frequencies of VH, VK, VL gene segment usage of 88 non-clonal sequences of isolated spike protein polyreactive antibodies from mRNA-LNP immunized macaques. (B) Frequencies of VH, VK, VL CDRH3 length of 88 non-clonal sequences of isolated spike protein reactive antibodies from mRNA-LNP immunized macaques. (C) Distribution of mutation rates for heavy and light chains of 88 non-clonal sequences of isolated spike protein reactive antibodies from mRNA-LNP immunized macaques. (D) Immunogenetic analysis of the monoclonal antibodies isolated from wild-type spike mRNA-LNP vaccination in rhesus macaques.

	Antigens																		
Antibody ID	SARS-CoV-2 (2019-nCoV) Spike Protein (S1+S2 ECD)	SARS-CoV-2 Spike protein (RBD)	SARS-CoV-2 (2019-nCoV) Spike Protein (S2 ECD)	SARS-CoV Spike Protein DeltaTM Recombinant	SARS-CoV, WH20 Coronavirus spike S1	SARS-CoV-1 RBD	MERS-CoV Coronavirus spike RBD	MERS-CoV Coronavirus spike S2	MERS-CoV, Coronavirus spike S1+S2	Recombinant MERS-CoV Spike/S1 Protein (S1 subunit)	HCoV-NL63 Spike Protein (S1+S2 ECD)	HCoV-229E Spike Protein (S1+S2 ECD)	Human Coronavirus HKU1 (isolate N5) (HCoV-HKU1) Spike Protein (S1+S2 ECD)	HCoV-OC43 Spike Protein (S1+S2 ECD)	nCOV-1 nCOV-2p	NTD	Streptavidin control	biotin-Man9 V3 (negative control)	Candida Albicans
DH1323	0.204	0.902	0.003	0.031	-0.020	-0.003	0.195	-0.031	-0.004	-0.008	-0.020	-0.017	-0.009	-0.012	-0.029	0.005	0.000	0.021	0.011
DH1324	0.009	0.109	0.023	0.001	-0.014	0.015	-0.027	-0.028	-0.008	-0.009	-0.033	-0.025	-0.022	-0.032	-0.046	-0.009	0.000	-0.032	0.073
DH1325	2.611	3.303	0.032	0.075	0.726	0.188	-0.014	-0.018	0.006	0.003	-0.008	-0.024	-0.012	-0.011	0.257	0.002	0.000	-0.020	0.052
DH1326	2.930	3.327	0.022	0.533	2.780	3.229	0.004	0.002	0.019	0.009	-0.001	-0.006	0.025	0.012	0.423	0.168	0.000	0.005	0.047
DH1327	3.013	3.260	0.039	-0.004	0.011	0.007	-0.021	-0.018	-0.003	-0.009	-0.018	-0.024	-0.006	-0.012	0.336	0.002	0.000	-0.004	0.009
DH1328	-0.007	0.008	-0.010	-0.008	-0.004	0.019	-0.017	-0.019	0.004	0.031	-0.030	-0.026	-0.007	-0.004	-0.072	-0.053	0.000	-0.012	0.007
DH1329	3.287	3.507	0.003	0.126	0.558	0.721	-0.003	0.007	0.007	0.012	0.029	0.009	0.031	0.029	1.059	0.036	0.000	-0.049	0.003
DH1330	3.089	3.502	0.035	0.025	0.092	0.291	-0.021	-0.007	-0.006	0.005	0.013	-0.005	-0.009	-0.002	0.705	0.095	0.000	-0.045	-0.015
DH1331	2.455	3.384	-0.006	0.000	0.257	2.085	-0.002	-0.004	-0.003	0.010	0.011	0.012	0.007	0.006	0.369	0.058	0.000	-0.026	0.033
DH1332	2.820	3.446	0.279	-0.016	0.005	0.067	0.168	0.040	-0.002	0.001	0.002	-0.014	0.035	-0.004	0.300	0.087	0.000	-0.036	0.024
DH1333	2.945	3.410	0.085	0.077	0.144	0.144	0.080	0.133	0.082	0.080	0.094	0.127	0.126	0.120	0.873	0.011	0.000	-0.142	-0.355
DH1334	2.077	3.080	0.004	0.283	2.204	2.448	-0.011	-0.004	0.002	0.024	0.021	-0.014	-0.014	-0.003	0.500	1.856	0.000	-0.040	0.002
DH1335	2.990	3.449	0.105	1.181	3.188	3.360	-0.002	0.039	0.041	0.006	0.017	0.019	0.030	0.031	0.465	1.005	0.000	-0.043	0.046
DH1336	0.305	0.967	0.032	-0.012	-0.014	0.038	-0.006	0.002	0.005	-0.001	-0.003	-0.006	-0.006	-0.016	0.087	0.073	0.000	-0.041	-0.016
DH1337	2.591	3.228	0.043	1.055	2.941	3.190	-0.012	0.030	0.024	0.010	0.027	0.035	0.065	0.079	0.284	0.298	0.000	-0.128	-0.083
DH1338	2.846	3.451	0.019	1.981	3.346	3.495	0.025	0.022	0.023	0.029	0.067	0.027	0.043	0.031	0.624	0.012	0.000	-0.014	0.534
DH1339	0.002	0.088	0.052	0.005	-0.011	0.039	0.009	0.009	0.049	0.012	0.010	0.010	0.025	0.024	-0.013	0.109	0.000	-0.045	0.141
DH1340	2.981	3.196	0.053	0.017	0.120	0.133	0.049	0.022	0.044	0.043	0.049	0.028	0.052	0.055	0.588	0.340	0.000	-0.078	0.353
DH1341	3.028	3.378	-0.003	0.170	1.678	1.598	0.006	0.003	0.020	0.013	0.026	0.023	0.025	0.025	0.689	0.019	0.000	-0.069	0.132
DH1342	1.539	3.233	0.060	0.008	0.038	0.035	0.034	0.050	0.019	0.028	0.032	0.044	0.045	0.019	0.196	0.080	0.000	-0.031	0.027
DH1343	2.610	3.301	0.006	-0.005	-0.028	0.028	0.021	0.010	0.032	0.046	0.036	0.009	0.041	0.060	0.479	0.595	0.000	0.060	0.275
DH1344	2.345	3.344	0.043	0.033	0.005	0.063	0.050	0.045	0.052	0.117	0.181	0.089	0.062	0.036	0.960	-0.026	0.000	0.023	-0.081
DH1345	3.155	3.490	3.144	0.040	0.152	0.263	0.040	0.057	0.133	0.028	0.028	0.044	0.054	0.036	0.543	0.497	0.000	-0.063	0.212
DH1453	3.125	0.032	0.005	0.924	3.105	0.011	-0.011	-0.002	-0.014	0.004	0.002	0.003	0.009	0.009	0.815	3.366	0.000	-0.037	-0.013
DH1454	1.297	0.047	-0.008	0.064	1.141	0.037	-0.033	0.004	0.029	-0.006	0.022	-0.002	-0.022	-0.016	0.084	3.216	0.000	-0.009	0.080
DH1455	0.034	-0.004	-0.012	-0.001	-0.036	0.004	-0.011	0.007	-0.002	0.009	0.034	0.013	0.012	0.017	-0.022	0.359	0.000	-0.033	-0.011
DH1456	1.972	0.355	0.017	0.004	0.020	0.052	0.004	0.009	0.025	0.017	0.021	0.013	0.025	0.021	0.289	3.077	0.000	-0.066	0.011
DH1457	0.124	0.134	0.007	-0.018	-0.029	0.002	-0.012	0.001	0.001	0.000	-0.003	0.000	0.005	0.038	0.035	0.772	0.000	-0.174	-0.150
DH1458	1.476	0.070	-0.013	0.051	0.035	0.147	-0.006	0.026	0.031	0.006	0.003	0.004	0.016	0.017	0.689	3.289	0.000	-0.007	-0.024
DH1459	0.188	0.275	0.019	0.044	0.243	0.278	-0.012	0.010	0.004	0.003	0.007	0.004	-0.001	0.022	-0.026	0.009	0.000	-0.036	-0.001
DH1460	0.009	0.100	-0.023	-0.017	-0.058	0.023	0.012	0.025	0.013	0.002	0.004	0.009	-0.016	-0.002	0.123	2.720	0.000	-0.262	-0.244
DH1461	-0.003	0.037	-0.032	-0.019	-0.058	0.003	0.010	-0.011	-0.017	0.000	-0.006	-0.009	-0.019	0.000	0.428	3.298	0.000	-0.020	0.012
DH1462	1.962	0.046	0.361	0.000	0.019	0.052	0.020	0.022	0.023	0.011	0.012	0.014	0.027	0.035	0.546	3.282	0.000	-0.018	0.023
DH1463	0.530	1.658	-0.011	-0.020	0.097	0.146	-0.020	0.000	0.002	0.005	-0.009	-0.004	-0.023	-0.015	0.973	3.299	0.000	-0.035	-0.013
DH1464	0.785	0.023	0.033	0.005	-0.022	0.021	-0.007	-0.005	0.001	0.009	0.003	0.004	0.010	0.006	0.557	3.111	0.000	0.001	0.106
DH1465	0.066	0.052	0.010	-0.012	-0.011	0.009	0.001	0.041	0.042	0.010	0.000	0.029	0.025	0.031	0.487	3.211	0.000	-0.027	-0.004
DH1466	0.029	0.002	-0.014	-0.004	0.009	0.004	-0.027	-0.007	-0.005	0.002	-0.002	-0.003	-0.019	-0.015	0.337	1.887	0.000	-0.254	-0.252
DH1467	0.015	0.014	-0.009	0.005	-0.001	0.012	-0.006	-0.019	-0.014	0.002	0.010	0.031	-0.011	0.025	-0.041	0.334	0.000	0.010	0.033
DH1468	2.862	3.584	0.106	0.091	0.071	0.145	0.097	0.075	0.117	0.099	0.066	0.104	0.107	0.059	0.538	-0.069	0.000	-0.206	0.100
DH1469	2.929	3.431	0.005	0.013	-0.021	0.043	-0.011	0.008	0.005	0.001	-0.003	0.000	-0.014	-0.014	0.530	0.026	0.000	-0.038	0.007
DH1470	2.718	3.305	-0.024	-0.018	0.022	0.003	-0.023	-0.008	-0.024	0.004	-0.004	-0.012	-0.018	-0.015	0.511	0.727	0.000	-0.056	-0.026
DH1471	1.209	2.813	0.312	0.055	0.128	0.067	0.105	0.076	0.080	0.073	0.048	0.164	0.288	0.081	-0.015	0.159	0.000	0.005	0.316
DH1472	1.810	3.171	-0.005	-0.003	0.009	0.011	-0.018	-0.018	-0.003	-0.002	-0.024	-0.014	-0.011	-0.003	0.120	-0.001	0.000	-0.001	-0.002
DH1473	0.128	0.159	-0.003	0.015	0.108	0.176	-0.002	0.043	0.024	0.039	0.000	0.029	0.041	0.049	0.133	0.068	0.000	-0.024	0.009
DH1474	1.070	2.250	0.043	0.007	0.005	0.058	0.038	0.052	0.043	0.022	0.020	0.025	0.086	0.054	0.206	0.245	0.000	-0.093	-0.081
DH1475	2.304	3.481	0.009	1.238	3.268	3.442	-0.014	-0.027	-0.006	-0.004	-0.014	0.054	-0.017	-0.017	0.117	0.003	0.000	-0.015	0.015
DH1476	2.878	3.335	-0.010	1.509	3.134	-0.001	-0.014	-0.008	0.000	0.007	-0.023	-0.021	0.356	-0.004	0.198	0.007	0.000	0.052	0.044
DH1477	0.629	0.040	0.746	0.089	-0.006	0.021	0.025	0.027	0.026	0.030	0.015	0.063	0.041	0.026	0.487	-0.005	0.000	-0.053	0.160
DH1478	3.228	0.755	3.374	0.169	0.230	0.235	0.008	0.041	0.171	0.063	0.048	0.075	0.109	0.106	0.178	0.256	0.000	-0.212	0.768
DH1479	-0.023	0.007	-0.024	-0.017	-0.058	0.004	0.004	0.013	0.003	0.001	-0.011	-0.012	-0.008	-0.004	0.011	2.103	0.000	-0.024	0.004
DH1480	0.372	0.370	0.329	0.220	0.304	0.346	0.413	0.307	0.265	0.245	0.246	0.269	0.340	0.344	0.037	1.689	0.000	0.228	0.138
DH1481	0.805	0.093	0.048	0.068	0.118	0.113	0.051	0.031	0.088	0.044	0.049	0.084	0.091	0.056	0.076	1.939	0.000	0.014	0.104
DH1482	2.712	3.302	-0.002	0.400	2.358	0.008	-0.019	0.001	0.001	0.024	0.010	0.028	0.011	0.031	1.034	3.721	0.000	-0.016	0.126
DH1483	2.056	3.171	-0.011	0.039	-0.016	-0.002	-0.008	-0.017	-0.005	-0.001	-0.029	-0.032	-0.012	-0.008	0.118	0.015	0.000	-0.006	0.017
DH1484	2.459	3.100	0.004	0.005	-0.057	0.034	-0.021	0.013	0.005	0.000	-0.006	-0.009	0.007	0.006	0.388	0.042	0.000	-0.057	0.025
DH1485	2.519	3.158	0.019	0.002	0.000	0.044	-0.023	-0.027	-0.010	0.010	-0.027	-0.032	0.039	0.018	0.157	0.045	0.000	0.008	0.025
DH1486	-0.020	0.000	-0.003	0.019	0.040	0.015	-0.011	0.033	-0.005	-0.004	-0.008	-0.006	-0.008	-0.012	-0.025	0.102	0.000	-0.032	0.024
DH1487	0.125	0.064	0.010	0.002	-0.014	0.033	0.015	0.019	0.006	0.000	-0.004	-0.004	0.004	0.004	0.056	2.179	0.000	-0.041	0.000

Figure S6. High-throughput ELISA binding screen of antibodies recovered from wildtype SARS-CoV-2 spike mRNA-LNP vaccinated rhesus macaques. Antibodies were tested in unpurified cell culture supernatant at a single dilution. Yellow highlighted values are above 0.1 OD450nm and are considered positive.

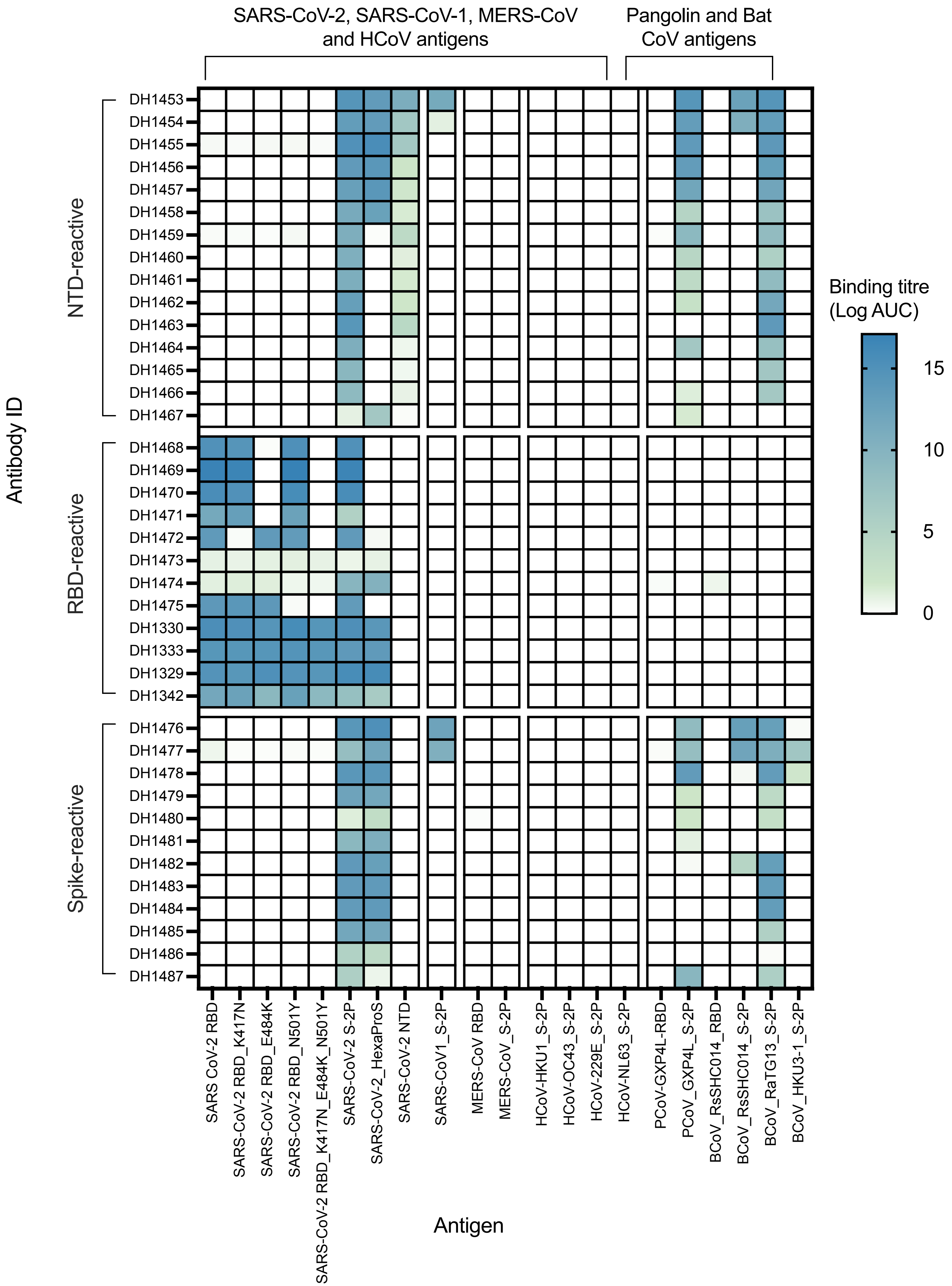

SARS-CoV-2, SARS-CoV-1, MERS-CoV and HCoV antigens
Pangolin and Bat CoV antigens
Antibody ID
NTD-reactive
RBD-reactive
Spike-reactive
DH1453
DH1454
DH1455
DH1456
DH1457
DH1458
DH1459
DH1460
DH1461
DH1462
DH1463
DH1464
DH1465
DH1466
DH1467
DH1468
DH1469
DH1470
DH1471
DH1472
DH1473
DH1474
DH1475
DH1330
DH1333
DH1329
DH1342
DH1476
DH1477
DH1478
DH1479
DH1480
DH1481
DH1482
DH1483
DH1484
DH1485
DH1486
DH1487
SARS CoV-2 RBD
SARS-CoV-2 RBD_K417N
SARS-CoV-2 RBD_E484K
SARS-CoV-2 RBD_N501Y
SARS-CoV-2 RBD_K417N_E484K_N501Y
SARS-CoV-2 S-2P
SARS-CoV-2_HexaProS
SARS-CoV-2 NTD
SARS-CoV1_S-2P
MERS-CoV RBD
MERS-CoV_S-2P
HCoV-HKU1_S-2P
HCoV-OC43_S-2P
HCoV-229E_S-2P
HCoV-NL63_S-2P
PCoV-GXP4L-RBD
PCoV_GXP4L_S-2P
BCoV_RsSHC014_RBD
BCoV_RsSHC014_S-2P
BCoV_RaTG13_S-2P
BCoV_HKU3-1_S-2P
Antigen
Binding titre (Log AUC)
15
10
5
0

Figure S7. Monoclonal antibodies isolated from wildtype S-tm mRNA-LNP immunized rhesus macaques bind SARS-CoV-2 variants and SARS-related betacoronaviruses and possess different epitope specificities. Antibodies labeled Spike-reactive bound to various domains of Spike in initial binding screens and confirmatory ELISA.

A

Competitive relationship between vaccine-induced rhesus macaque mAbs with vaccine or infection induced human/ mouse mAbs

		DH1041	DH1042	DH1044	DH1047	DH1073	DH1235	DH1284	DH1193	S309	CR3022	SP1-77
Outer RBD face macaque antibodies	DH1333	1.322	1.223	0.508	10.000	10.000	10.000	10.000	0.329	10.000	10.000	1.014
	DH1345	10.000	10.000	0.425	10.000	8.864	10.000	10.000	0.208	10.000	10.000	10.000
	DH1341	10.000	0.605	0.309	10.000	1.223	10.000	10.000	0.231	0.985	10.000	10.000
	DH1344	10.000	10.000	0.562	10.000	10.000	10.000	10.000	0.248	10.000	10.000	10.000
	DH1343	10.000	10.000	0.685	10.000	10.000	10.000	10.000	0.326	10.000	10.000	10.000
	DH1329	0.417	0.421	0.214	10.000	10.000	10.000	10.000	10.000	0.522	10.000	1.021
	DH1330	0.944	0.705	0.334	10.000	10.000	10.000	10.000	0.214	10.000	10.000	10.000
	DH1325	10.000	10.000	0.501	10.000	10.000	10.000	10.000	0.287	10.000	10.000	10.000
	DH1340	10.000	10.000	0.569	10.000	4.369	10.000	10.000	0.300	10.000	10.000	1.200
	DH1335	10.000	10.000	0.537	10.000	10.000	10.000	10.000	0.224	10.000	10.000	10.000
Inner RBD face macaque antibodies	DH1338	10.000	10.000	10.000	0.346	10.000	0.215	10.000	10.000	10.000	0.086	10.000
	DH1328	10.000	10.000	10.000	0.384	10.000	0.217	10.000	10.000	10.000	0.104	10.000
	DH1337	10.000	10.000	10.000	0.782	10.000	0.676	10.000	10.000	10.000	0.236	10.000
	DH1324	10.000	10.000	10.000	0.539	10.000	0.475	10.000	10.000	10.000	0.237	10.000
	DH1336	10.000	10.000	10.000	1.380	10.000	10.000	10.000	10.000	10.000	0.171	10.000
	DH1326	10.000	10.000	10.000	1.087	10.000	0.528	10.000	10.000	10.000	0.183	10.000
	DH1339	10.000	10.000	10.000	4.504	10.000	3.078	10.000	10.000	10.000	0.981	10.000
	DH1331	10.000	10.000	10.000	10.000	10.000	10.000	10.000	10.000	10.000	0.260	10.000
	DH1327	10.000	10.000	10.000	10.000	10.000	10.000	10.000	10.000	10.000	2.244	10.000
	DH1332	10.000	10.000	10.000	10.000	10.000	10.000	10.000	10.000	10.000	10.000	10.000
	DH1334	10.000	10.000	10.000	10.000	10.000	10.000	10.000	10.000	10.000	10.000	10.000
	DH1342	10.000	10.000	10.000	10.000	10.000	10.000	10.000	10.000	10.000	10.000	10.000

IC_{50} (ug/mL): <0.1 | <1 | <10 | <5 | >10

B

	Live virus neutralization			Pseudovirus neutraliazation (FDA)									Psedovirus neutraliazation (Duke)	
	SARS-CoV-2 D614G	SARS-CoV	RsSHC014	WA-1	D614G	B.1.1.7 (Alpha)	B.1.351 (Beta)	P.1 (Gamma)	B.1.617.2 (Delta)	B.1.617.1 (Kappa)	B.1.429 (Epsilon)	B.1.526 (Iota)	BA.1 (Omicron)	BA.4/BA.5
DH1333	0.006	10.000	10.000	0.016	0.010	0.030	0.022	0.028	0.025	0.016	0.016	0.020	50.000	0.030
DH1345	1.231	10.000	10.000	0.141	0.660	0.157	0.611	0.177	0.315	0.326	0.156	0.515	1.600	3.400
DH1341	6.368	10.000	10.000	1.401	3.600	1.481	3.300	1.658	2.924	1.647	1.304	2.755	3.300	12.000
DH1344	0.220	10.000	10.000	0.444	3.900	0.572	1.374	0.654	0.951	1.414	0.560	1.112	28.000	21.000
DH1343	1.107	10.000	10.000	0.533	2.500	0.588	1.255	0.589	0.913	1.127	0.621	1.443	18.000	25.000
DH1329	0.003	10.000	10.000	0.023	0.010	0.023	0.022	0.024	10.000	10.000	10.000	0.027	0.280	50.000
DH1330	0.206	10.000	10.000	0.120	0.330	0.143	2.793	0.538	10.000	10.000	10.000	0.904	4.300	50.000
DH1325	2.335	10.000	10.000	2.695	10.000	4.717	10.000	10.000	10.000	10.000	2.994	10.000	50.000	50.000
DH1340	10.000	10.000	10.000	10.000	10.000	10.000	10.000	10.000	10.000	10.000	10.000	10.000	26.000	50.000
DH1335	10.000	10.000	10.000	10.000	10.000	10.000	10.000	10.000	10.000	10.000	10.000	10.000	41.000	50.000
DH1338	0.020	0.425	0.307	0.061	0.040	0.067	0.067	0.062	0.064	0.063	0.061	0.060	40.000	50.000
DH1328	0.321	10.000	1.081	2.041	0.560	1.957	3.236	3.040	1.931	2.083	1.815	2.933	25.000	50.000
DH1337	0.202	2.972	1.178	0.602	0.320	0.664	0.716	0.716	0.561	0.671	0.652	0.676	50.000	50.000
DH1324	0.160	10.000	10.000	0.613	0.650	0.880	0.917	0.908	0.620	0.759	0.788	0.879	50.000	50.000
DH1336	0.454	10.000	10.000	0.536	0.790	0.807	1.272	1.458	0.551	0.843	0.577	1.311	50.000	50.000
DH1326	1.587	10.000	10.000	1.299	0.760	1.460	2.004	1.669	1.350	1.748	1.689	2.169	50.000	50.000
DH1339	10.000	10.000	10.000	10.000	10.000	10.000	10.000	10.000	10.000	10.000	10.000	10.000	50.000	50.000
DH1331	10.000	10.000	10.000	10.000	10.000	10.000	10.000	10.000	10.000	10.000	10.000	10.000	50.000	50.000
DH1327	10.000	10.000	10.000	10.000	10.000	10.000	10.000	10.000	10.000	10.000	10.000	10.000	50.000	50.000
DH1332	0.601	10.000	10.000	0.674	10.000	2.370	10.000	2.809	10.000	10.000	10.000	10.000	50.000	50.000
DH1334	10.000	10.000	10.000	10.000	10.000	10.000	10.000	10.000	10.000	10.000	10.000	10.000	50.000	50.000
DH1342	10.000	10.000	10.000	10.000	10.000	10.000	10.000	10.000	10.000	10.000	10.000	10.000	50.000	50.000

<0.01 | <0.1 | <1 | <10 | >10

<0.01 | <0.1 | <1 | <10 | <50 | >50

C

		BA.4/BA.5	BA.4.6	BA.2.75.2	BF.7	BQ.1.1	XBB.1	XBB1.5
Outer RBD face macaque antibodies	DH1333	0.030	50.000	50.000	50.000	50.000	50.000	50.000
	DH1345	3.400	1.700	6.300	1.500	4.400	10.000	8.500
	DH1341	12.000	7.100	6.500	4.300	5.700	5.300	10.000
	DH1344	21.000	11.000	11.000	11.000	13.000	43.000	20.000
	DH1343	25.000	7.100	13.000	4.900	6.100	20.000	12.000
	DH1329	50.000	50.000	50.000	50.000	50.000	50.000	50.000
	DH1340	50.000	50.000	50.000	29.000	50.000	20.000	34.000
	DH1335	50.000	50.000	47.000	50.000	50.000	18.000	39.000
	DH1330	50.000	50.000	50.000	50.000	50.000	50.000	50.000
Inner RBD face macaque antibodies	DH1338	50.000	50.000	50.000	50.000	50.000	50.000	50.000
	DH1328	50.000	45.000	50.000	35.000	50.000	39.000	50.000

IC_{50} (ug/mL): <0.01 | <0.1 | <1 | <10 | <50 | >50

Figure S8. Distinct antibody clones confer pan-SARS-CoV-2 variants of concern neutralization or Sarbecovirus cross-neutralization. (A) Vaccine-induced macaque antibody concentration required to block 50% of human antibody binding to Spike. (B,C) IC50 neutralization titer for monoclonal antibodies isolated from wild-type mRNA-LNP vaccinated rhesus macaques. Titers are shown for replicating SARS-CoV-2, SARS-CoV, RsSCHC014 viruses, and SARS-CoV-2 pseudovirus variants of concern. A subset of antibodies were tested against pseudoviruses of SARS-CoV-2 Omicron sublineages (BA.4/BA.5, BA.4.6, BA2.75.2, BF.7, BQ.1.1, XBB.1 AND XBB1.5) variants in 293T-ACE2 cells.

Supplementary figure 9

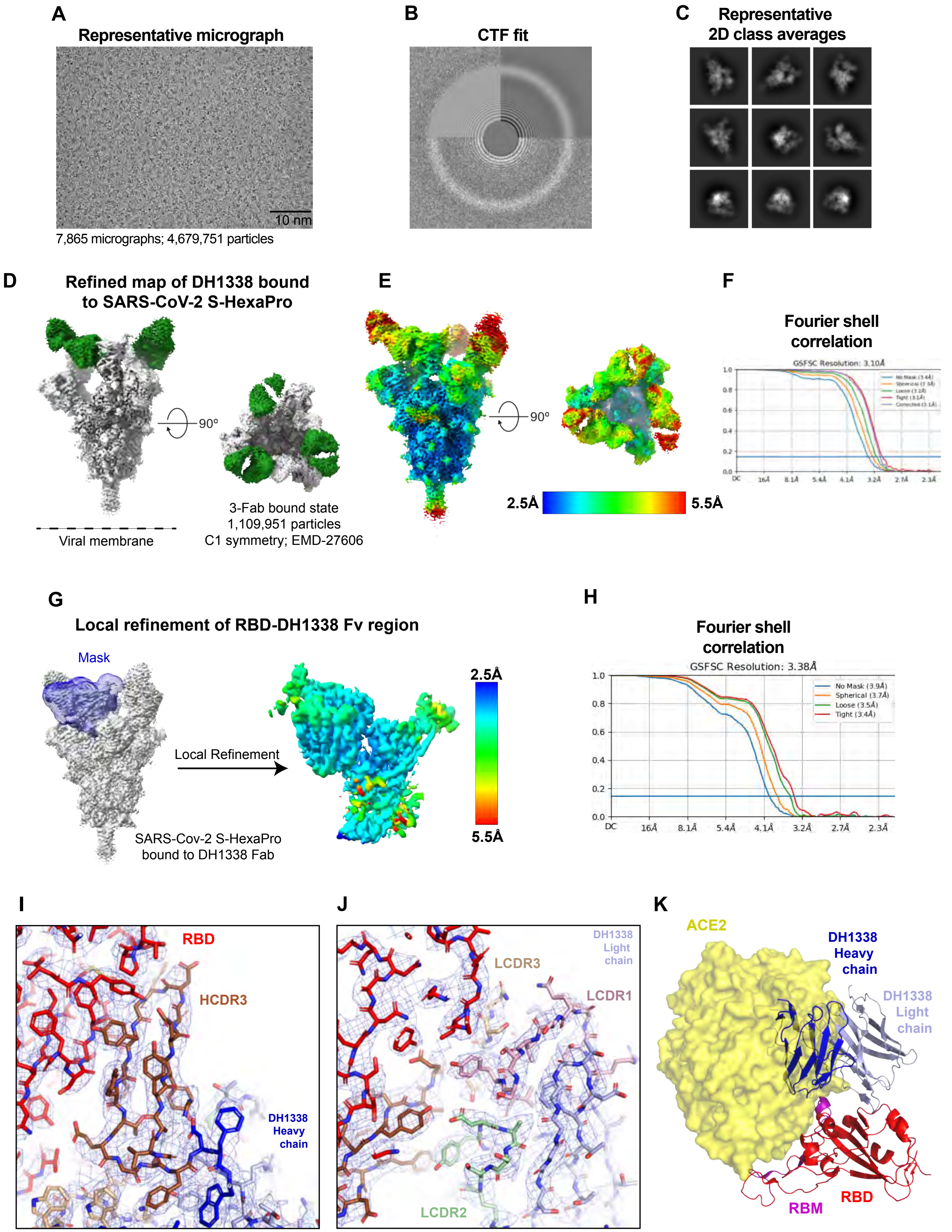

Figure S9. Cryo-EM data processing for Antibody DH1338 in complex with SARS-CoV-2 S protein, Related to Figure Cryo-EM-1. (A) Representative micrograph. (B) Representative CTF fit. (C) Representative 2D class averages from cryo-EM dataset. Box size = 324 Å. (D) Refined 3D map segmented and colored by component, with the SARS-CoV-2 S-HexaPro protein colored in grey and DH1338 colored green. (E) Refined map shown in D colored by local resolution. (F) Fourier Shell Correlation (FSC) curves of the 3D reconstruction shown in D with horizontal blue line indicating FSC 0.143. (G) Local refinement of RBD-DH1338 Fv region. Left Blue mesh shows the mask that was used for local refinement. Right. Local Refined 3D density map colored by local resolution. (H) FSC curves of local refined map shown in G. (I) DH1338 HC binding interface with RBD, shown in sticks with electron density shown in blue mesh. (J) DH1338 LC binding interface with RBD. PDBID: 8DPZ. (K) ACE2 (yellow surface representation; PDB 6M0J) binding to RBD (Red cartoon with RBM shown in purple; PDB 6M0J) is sterically hindered by DH1338 (Blue, HC; light blue, LC; cartoon representation; PDB 8DPZ).

Supplementary figure 10

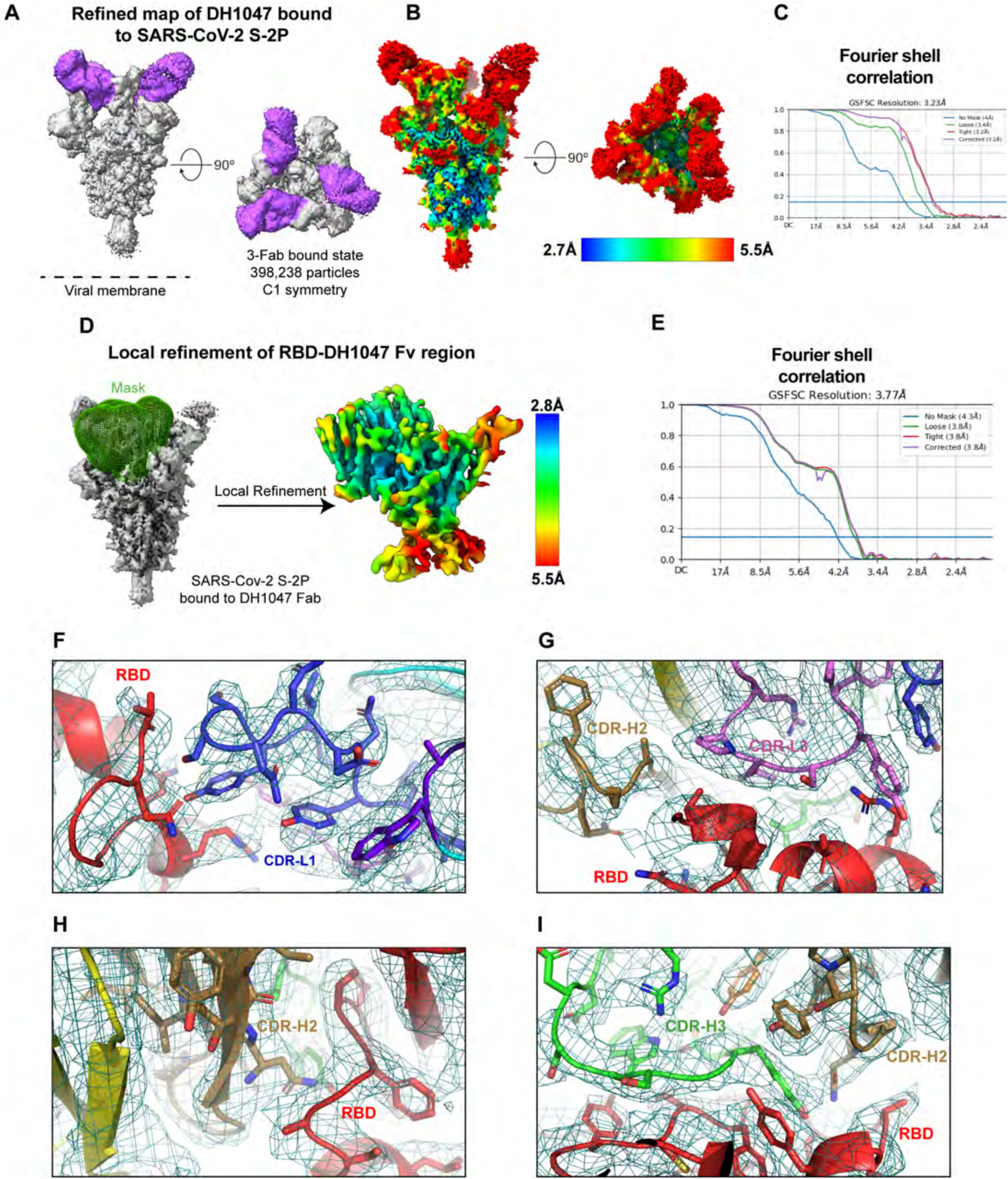

Figure S10. Cryo-EM data processing for Antibody DH1047 in complex with SARS-CoV-2 S protein. (A) Refined map of the SARS-CoV-2 2P spike bound to three DH1047 Fabs, highlighted in violet. (B) The map from A. colored by local resolution. (C) Fourier Shell Correlation for the refinement of the full spike-Fab complex. (D) Local refinement region covered by the mask shown in green mesh and resulting map from local refinement, colored by local resolution. The resulting map included the spike RBD and Fv region of DH1047 (E) Fourier Shell Correlation of the locally refined map. (F-I) Representative views of the fit of the model to the local refinement map. (F) CDR-L1 to RBD contact. (G) CDR-L3 and H2 to RBD contact. (H) CDR-H2 to RBD contact. (I) CDR-H2 and H3 to RBD contact.

Table S1. Detailed interactions of DH1338 HC with RBD from Pisa Server.

	Residue	HSDC	BSA	
DH1338 Heavy chain	F:ARG 31	H	69.03	|||||
	F:SER 53		10.41	||
	F:GLU 99	H	19.12	||
	F:ASP 100		1.23		
	F:ASP 100A	H	35.04	|||
	F:TYR 100B	H	99.75	|||||
	F:GLY 100C		39.88	||||||||||
	F:TYR 100D	H	121.83	||||||||||
	F:TYR 100E		48.20	||||
	F:TYR 100F	H	31.61	|||||
	F:GLU 100H	H	65.90	||||
RBD	B:TYR 369	H	68.21	||||||||
	B:ASN 370		18.17	||
	B:SER 371	H	16.90	||||
	B:ALA 372		44.44	||||
	B:PHE 374		23.44	||||||
	B:SER 375	H	59.37	||||||||
	B:THR 376	H	20.93	|||||||
	B:PHE 377	H	41.65	|||||||||
	B:LYS 378		39.03	||||
	B:CYS 379	H	29.48	||||||||||
	B:TYR 380		2.98		
	B:GLY 381		22.73	|||
	B:VAL 382		5.28	||
	B:SER 383	H	35.93	||||||
	B:PRO 384		26.83	||||||||||
	B:THR 385		4.82		
	B:ASN 437		3.14		
	B:VAL 503		30.77	||||
	B:TYR 508	H	13.46	||||||

HSDC: Residues making Hydrogen/Disulphide bond, Salt bridge or Covalent link, BSA: Buried Surface Area, Å2. |||| Buried area percentage, one bar per 10%.

Table S2. Detailed interactions of DH1338 LC with RBD from Pisa Server.

<table>
<tr><th></th><th>Residue</th><th>HSDC</th><th>BSA</th><th></th></tr>
<tr><td rowspan="5">DH1338 Light chain</td><td>G:SER 28</td><td></td><td>11.51</td><td>||</td></tr>
<tr><td>G:SER 30</td><td>H</td><td>57.51</td><td>|||||||||</td></tr>
<tr><td>G:SER 31</td><td></td><td>19.40</td><td>|||||</td></tr>
<tr><td>G:TYR 32</td><td>H</td><td>69.75</td><td>|||||||</td></tr>
<tr><td>G:SER 67</td><td></td><td>0.25</td><td>|</td></tr>
<tr><td rowspan="11">RBD</td><td>B:ARG 403</td><td></td><td>0.58</td><td>|</td></tr>
<tr><td>B:GLY 404</td><td>H</td><td>10.12</td><td>||||||</td></tr>
<tr><td>B:ASP 405</td><td>H</td><td>39.62</td><td>||||||</td></tr>
<tr><td>B:ARG 408</td><td></td><td>8.58</td><td>|</td></tr>
<tr><td>B:THR 500</td><td></td><td>11.05</td><td>|</td></tr>
<tr><td>B:GLY 502</td><td></td><td>32.48</td><td>||||||||</td></tr>
<tr><td>B:VAL 503</td><td>H</td><td>59.62</td><td>|||||||</td></tr>
<tr><td>B:GLY 504</td><td></td><td>26.08</td><td>||||||||||</td></tr>
<tr><td>B:TYR 505</td><td></td><td>25.69</td><td>|||</td></tr>
<tr><td>B:GLN 506</td><td></td><td>8.88</td><td>||||</td></tr>
<tr><td>B:TYR 508</td><td></td><td>1.52</td><td>|</td></tr>
</table>

HSDC: Residues making Hydrogen/Disulphide bond, Salt bridge or Covalent link, BSA: Buried Surface Area, Å2. |||| Buried area percentage, one bar per 10%.

Table S3. DH1047-Bound SARS-CoV-2 S-2P: Local Refinement of RBD/Fab interface.

Cryo-EM data collection and refinement statistics.	
**Structure Name**	**DH1047-Bound SARS-CoV-2 S-2P: Local Refinement of RBD/Fab interface**
**PDB ID**	8DTK
**EMDB ID**	EMD-27703
**Data Collection and processing**	
Microscope	FEI Titan Krios
Detector	Gatan K3
Magnification	81000
Voltage (kV)	300
Electron exposure (e-/Å^2)	66.77
Defocus Range (μm)	~0.75-2.50
Pixel size (Å)	1.058
Reconstruction software	cryoSPARC
Symmetry imposed	C1
Initial particle images (no.)	2,797,281
Final particle images (no.)	398,238
Map resolution (Å)	3.77
FSC threshold	0.143
**Model composition**	
Nonhydrogen atoms	6942
Protein residues	449
**R.M.S. deviations**	
Bond lengths (Å)	0.005
Bond angles (°)	1.133
**Validation**	
MolProbity score	1.53
Clashscore	4.32
Rotamer Outliers (%)	0
**Ramachandran plot**	
Favored regions (%)	95.49
Allowed (%)	4.51
Disallowed regions (%)	0
